# Genome-Wide Characterization of the *PLATZ* Gene Family in *Medicago truncatula* and Their Expression Profiles in Response to Abiotic Stress

**DOI:** 10.3390/biology15151318

**Published:** 2026-08-06

**Authors:** Wen Zhou, Shuangjin Fan, Xinyan Zhao, Chenxi Zang, Le Wang, Yongwang Sun

**Affiliations:** College of Agriculture and Biology, Liaocheng University, Liaocheng 252000, China; 15553352126@163.com (W.Z.); 17865260368@163.com (S.F.); zxy5471099@163.com (X.Z.); 13375375749@163.com (C.Z.); 19853145479@163.com (L.W.)

**Keywords:** *Medicago truncatula*, PLATZ, gene family, expression pattern, abiotic stress

## Abstract

Plant A/T-rich sequence and zinc-binding protein (PLATZ) transcription factors are pivotal regulators of plant growth, development, and abiotic stress responses. Although *PLATZ* genes have been extensively studied and functionally characterized in various plant species, little information is available regarding these genes in the legume model plant *Medicago truncatula.* In this study, a total of 16 *PLATZ* genes (designated *MtPLATZ1* to *MtPLATZ16*) were identified from the *M. truncatula* genome and distributed across six of the eight chromosomes. Subcellular localization prediction analysis indicated that all MtPLATZ proteins are localized in the nucleus, with eight representative members experimentally validated by transient expression assays. Collinearity analysis indicated that five segmental duplication pairs were the primary driver of *MtPLATZ* gene expansion. Based on phylogenetic analysis, these genes were classified into four groups, comprising 6, 2, 4, and 4 genes, respectively. Gene structure analysis demonstrated that these genes possess three or four exons. Most *MtPLATZ* genes were found to contain at least one ABRE *cis*-element in their promoter regions, and a few also harbored LTR, CAT-box, ARE, and DRE *cis*-elements. Expression analysis revealed that *MtPLATZ* genes exhibit distinct expression patterns across different tissues, suggesting that they may be differentially regulated by abiotic stresses in a tissue-specific manner. Under PEG-induced osmotic stress, acute NaCl shock, acute heat shock, and acute cold shock, the expression levels of these eight genes exhibited significant changes, particularly *MtPLATZ2*, *MtPLATZ6*, and *MtPLATZ9*, indicating that *MtPLATZ* gene family members may play a broad role in abiotic stress responses. In summary, these findings provide valuable insights for future functional exploration of *MtPLATZ* genes and contribute to a deeper understanding of their roles in stress responses in *M. truncatula*.

## Simple Summary

PLATZ transcription factors are a plant-specific family of zinc finger proteins that play important roles in regulating plant growth, development, and abiotic stress responses. In the legume model plant *Medicago truncatula*, the *PLATZ* gene family has not been systematically characterized. In this study, a total of 16 *MtPLATZ* genes were identified in the *M. truncatula* genome and classified into four distinct subgroups based on phylogenetic analysis. This family includes five segmental duplication pairs and one tandem duplication pair, all of which have *K*a/*K*s ratios less than 1, indicating that *MtPLATZ* genes have experienced strong purifying selection during evolution. *Cis*-acting element analysis revealed that the promoter regions of *MtPLATZ* genes contain multiple stress-responsive and hormone-responsive elements, suggesting their potential extensive involvement in environmental adaptation. Preliminary expression profiling under various abiotic stresses, including PEG-induced osmotic stress, acute NaCl shock, acute heat shock, and acute cold shock, demonstrated that *MtPLATZ* genes exhibit treatment-specific and time-dependent expression patterns, indicating that they may have functional divergence. This study provides a systematic analysis of the *MtPLATZ* gene family in *M. truncatula*, offering a valuable reference for functional studies and genetic improvement of stress tolerance in legumes.

## 1. Introduction

Transcription factors (TFs) serve as key regulatory proteins that regulate gene expression through their binding to specific *cis*-acting elements located within promoter regions [1,2]. As vital transcriptional activators or repressors, TFs control a wide range of biological processes, including signal transduction, cellular morphogenesis, and responses to both biotic and abiotic stresses [3,4]. In plants, zinc-finger transcription factor families exhibit high diversity, consisting of subgroups such as ERF, DOF, WRKY, and PHD [5]. Among these subgroups, Plant A/T-rich sequence and zinc-binding proteins (PLATZs) form a plant-exclusive subclass of zinc-finger TFs, which are indispensable for plant growth, development, and stress acclimation. The first identified *PLATZ* gene, named *PLATZ1*, was isolated from pea (*Pisum sativum*) by means of yeast one-hybrid screening of a cDNA library [6]. A distinctive feature of *PLATZ1* is the presence of two widely separated zinc-binding sites, which are critical for DNA binding activity and are defined by two conserved motifs: C-x_2_-H-x_11_-C-x_2_-C-x_(4–5)_-C-x_2_-C-x_(3–7)_-H-x_2_-H (M1) and C-x_2_-C-x_(10–11)_-C-x_3_-C (M2), where “x” denotes any amino acid residue. From a biochemical perspective, PLATZ1 specifically interacts with A/T-rich DNA sequences and has been shown to function as a transcriptional repressor for genes such as *pra2* (which encodes a GTPase) and *petE* (which encodes plastocyanin). Subsequent research efforts have verified that PLATZ represents a unique category of plant-specific TFs [6,7].

Gene family analysis is regarded as a fundamental approach for deciphering the function, structure, and evolutionary traits of genes [8,9]. With the rapid accumulation of genomic data, the *PLATZ* gene family has been comprehensively identified at the genome-wide level across a wide range of plant species. The count of *PLATZ* genes differs substantially among embryophytes, ranging from around a dozen to several tens of members [10]. For example: 11 *PLATZ* genes have been detected in barley (*Hordeum vulgare*) [11], pepper (*Capsicum annuum*) [12], and ginkgo (*Ginkgo biloba*) [13]; 12 in *Arabidopsis thaliana* [14]; 15 in rice (*Oryza sativa*) [15,16]; 17 in maize (*Zea mays*) [17], apple (*Malus domestica* Borkh.) [18], and sorghum (*Sorghum bicolor*) [19]; 20 in tomato (*Solanum lycopersicum*) [20]; 29 in paleopolyploid soybean (*Glycine max*) [21]; and as many as 62 members in hexaploid wheat (*Triticum aestivum*) [22]. This wide variation in gene family sizes highlights the crucial role of polyploidization in promoting the expansion of the *PLATZ* gene family.

Since the first identification of *PLATZ1* in pea, *PLATZ* genes have garnered substantial research attention owing to their essential regulatory functions across diverse plant species. Up to now, a large number of *PLATZ* genes have been identified and functionally characterized, particularly in the model plant *Arabidopsis* and several economically important crops [23]. The functional roles of *PLATZ* genes in different plant species are elaborated in detail as follows: In maize, the semi-dominant negative mutant *floury3* (*fl3*) displays severe endosperm abnormalities, and map-based cloning revealed that *Fl3* encodes a PLATZ protein (ZmPLATZ12) [17]. *FL3* regulates tRNA/5S rRNA transcription through RPC53/TFC1 interaction, affecting flowering and endosperm development [7]. Additionally, *ZmPLATZ2* has been found to regulate starch synthesis through upregulating the expression of starch synthase genes, including *ZmSSI*, *ZmISA1*, and *ZmISA2* [24]. In upland cotton, *GhPLATZ1* enhances osmotic/salt tolerance in transgenic *Arabidopsis* via hormone signaling pathways [25]. Cotton *GhPLATZ01* and *GhPLATZ15* confer drought tolerance in transgenic tomato via enhanced water retention, membrane stability, and antioxidant capacity [26]. Rice *GL6* regulates grain size by promoting cell proliferation in young panicles [15]. Similarly, overexpression of rice *SG6* increases plant height and grain size/weight by activating genes involved in DNA replication and the cell cycle [27]. Moso bamboo (*Phyllostachys edulis*) *PhePLATZ1* and *PhePLATZ8* confer drought/salt tolerance when overexpressed in *Arabidopsis* [28].

Among the well-investigated members of the *Arabidopsis* PLATZ transcription factor family, multiple genes have been confirmed to be involved in mediating plant tolerance to abiotic stresses. *AtPLATZ1* (*At1g21000*), also denoted as *AIN1*, serves as a positive regulator in enhancing dehydration tolerance in vegetative tissues. Additionally, it participates in abscisic acid (ABA)-mediated inhibition of primary root elongation, which is achieved by modulating the homeostasis of reactive oxygen species (ROS) [29,30]. In contrast to *AtPLATZ1*, *AtPLATZ2* (*At1g76590*) acts as a negative regulator of seedling salt tolerance. Its regulatory role is realized through the direct suppression of the expression of *CBL4/SOS3* and *CBL10/SCaBP8*, two genes closely associated with salt tolerance [31]. Similarly, *AtPLATZ4* (*At1g32700*) is strongly induced by drought stress and ABA treatment. It enhances plant drought tolerance by repressing the expression of *PIP2;8*, a plasma membrane aquaporin. Notably, *PIP2;8* exerts a negative effect on drought tolerance by inhibiting stomatal closure [32]. Furthermore, *AtPLATZ7* (*At2g12646*), also named *RITF1*, functions redundantly with *AtPLATZ2* in plant adaptation to salt stress. It is also essential for root meristem growth mediated by RGF1 (Root Growth Factor 1) through the ROS signaling pathway [33].

As the third-largest family of flowering plants worldwide, Leguminosae includes a variety of agronomically important crops, such as soybean, peanut (*Arachis hypogaea*), chickpea (*Cicer arietinum*), and alfalfa (*Medicago sativa*). These leguminous crops serve as crucial sources of high-quality proteins and oils for both human and animal nutrition [34]. Beyond their nutritional value, legumes play a vital role in improving soil fertility through the establishment of symbiotic associations with nitrogen-fixing bacteria. This symbiosis reduces the reliance on synthetic fertilizers and contributes to the development of more sustainable agricultural practices. Within the Leguminosae family, *Medicago truncatula* (barrel medic) has become a prominent model organism for legume genetics and molecular biology research. Its advantages include a diploid genome, self-pollinating characteristics, and an efficient genetic transformation system [35]. To date, several transcription factor families in *M. truncatula* have been systematically identified and functionally characterized, including NAC [36], MYB [37], HD-ZIP [38], and C2H2 [39]. These studies have laid a solid genomic foundation for exploring gene regulatory mechanisms in legumes. Although PLATZ transcription factors are known to be involved in regulating plant development and stress responses across multiple species, the *PLATZ* gene family in *M. truncatula* has not been thoroughly investigated. *M. truncatula* not only serves as a model system for studying legume-specific biological processes such as nodulation and nitrogen fixation, but also represents an ideal reference for elucidating stress tolerance mechanisms in forage and legume crops—traits that are absent in non-legume model plants such as *Arabidopsis* and rice. The recent publication of the telomere-to-telomere genome sequence of the reference accession Jemalong A17 has provided an opportunity for comprehensive and accurate analysis of the *MtPLATZ* family [40]. In the present study, we systematically identified *MtPLATZ* genes and analyzed their phylogenetic relationships, gene structures, protein motifs, chromosomal distributions, and duplication events. Additionally, we explored the *cis*-regulatory elements within their promoter regions. We further examined the expression patterns of *MtPLATZ* genes in different tissues and under various abiotic stresses to infer their potential biological functions. This research establishes a fundamental framework for future functional studies aimed at deciphering the molecular mechanisms by which *MtPLATZ* genes regulate growth, development, stress adaptation, and other important agronomic traits in *M. truncatula*.

## 2. Materials and Methods

### 2.1. Identification of the PLATZ Genes in the Genome of M. truncatula

To identify *PLATZ* genes in the genome of *M. truncatula*, the genome assembly and annotation files of *M. truncatula* accession Jemalong A17 were retrieved from the figshare repository (https://doi.org/10.6084/m9.figshare.29665022) [40]. Two complementary approaches were adopted to screen putative PLATZ proteins in *M. truncatula*. First, the hidden Markov model (HMM) profile corresponding to the PLATZ domain (PF04640) was downloaded from the InterPro database (https://www.ebi.ac.uk/interpro/, accessed on 10 February 2026). This HMM profile was then used to search the *M. truncatula* protein dataset using HMMER 3.4 software (http://hmmer.org/, accessed on 10 February 2026), with an E-value cutoff set at <1 × 10^−5^. Second, protein sequences of known *PLATZ* family members from *Arabidopsis* [14] were downloaded from The Arabidopsis Information Resource (TAIR; https://www.arabidopsis.org/, accessed on 10 February 2026). These AtPLATZ protein sequences were used as query sequences for BLASTP (https://blast.ncbi.nlm.nih.gov/Blast.cgi, accessed on 10 February 2026) searches against the *M. truncatula* protein dataset, with the same E-value threshold (<1 × 10^−5^) applied to ensure consistency in candidate screening. All candidate PLATZ proteins identified through the above two methods were further validated to confirm the presence of the PLATZ domain, which was performed using the InterPro database.

### 2.2. Prediction of the Physicochemical Properties and Subcellular Localization of MtPLATZ Proteins

The physicochemical properties of the identified MtPLATZ proteins were predicted using the ProtParam tool available on the ExPASy server (https://web.expasy.org/protparam/, accessed on 13 February 2026). The parameters analyzed included molecular weight, theoretical isoelectric point (pI), instability index, aliphatic index, and grand average of hydropathicity (GRAVY)—all of which are key indicators for characterizing protein physical and chemical features. Separately, the subcellular localizations of these MtPLATZ proteins were predicted via the WoLF PSORT tool (https://wolfpsort.hgc.jp/, accessed on 10 February 2026), a specialized resource for forecasting protein subcellular distribution in different organisms.

### 2.3. Chromosomal Localization and Synteny Analysis of MtPLATZ Genes

The genomic coordinates of all identified *MtPLATZ* genes were extracted from the GFF3-formatted genome annotation file of *M. truncatula*. To conduct synteny analysis, a genome-wide BLAST search was performed to identify homologous gene pairs, with a stringent E-value threshold of <10^−10^ applied to guarantee the reliability of high-confidence matches. Subsequently, the MCScanX algorithm integrated within TBtools software [41] was employed to analyze the obtained homologous pairs, aiming to define syntenic blocks and deduce gene duplication events. For visualizing segmental duplications of *MtPLATZ* genes, a Circos plot was constructed using the Advanced Circos module in TBtools, where solid red lines were used to connect duplicated gene pairs. To evaluate the evolutionary pressures acting on *MtPLATZ* genes, the non-synonymous (*K*a) and synonymous (*K*s) substitution rates were calculated for each duplicated gene pair using the *K*a/*K*s Calculator tool embedded in TBtools. The *K*a/*K*s ratios were interpreted according to conventional evolutionary principles: a ratio greater than 1 indicates positive selection, a ratio less than 1 suggests purifying selection, and a ratio approximately equal to 1 is consistent with neutral evolution.

### 2.4. Construction of the Phylogenetic Tree of the PLATZ Proteins

To clarify the evolutionary relationships among PLATZ family members, a phylogenetic analysis was carried out involving *M. truncatula* and three other angiosperm species, namely *Arabidopsis*, rice, and maize. First, multiple sequence alignment of the 60 full-length PLATZ protein sequences was performed using ClustalX 2.1 (https://www.genome.jp/tools-bin/clustalw, accessed on 17 February 2026). Subsequently, the resulting sequence alignment was used as input to construct an unrooted phylogenetic tree using MEGA 11.0 software (http://www.megasoftware.net, accessed on 17 February 2026). The neighbor-joining method was employed for tree construction, with the following parameters configured to ensure the reliability of the analysis: 1000 bootstrap replications to evaluate the confidence of the tree topology, the *p*-distance model, uniform substitution rates among sites, and pairwise gap deletion.

### 2.5. Analysis of the Gene Structure, Conserved Motifs, and Cis-Elements in Promoter

The genomic structures of *MtPLATZ* genes were analyzed using the GFF3-formatted genome annotation file of *M. truncatula*, and the exon–intron organization of these genes was visualized with TBtools software. For promoter analysis, the promoter sequences of each *MtPLATZ* gene were extracted from the genome assembly, which were defined as the 2 kb region upstream of the translation start codon (ATG). Putative *cis*-acting regulatory elements within these promoter sequences were predicted via the PlantCARE database (https://bioinformatics.psb.ugent.be/webtools/plantcare/html/, accessed on 22 February 2026). To prioritize elements with distinct biological significance, a filtering process was conducted to retain elements related to hormone responsiveness and stress induction. Specifically, elements associated with responsiveness to hormones such as abscisic acid (ABA), methyl jasmonate (MeJA), and salicylic acid (SA) were kept, as well as those linked to stress responses (e.g., drought, low temperature, and pathogen infection). In contrast, *cis*-acting elements categorized as miscellaneous or with unknown functions were excluded from subsequent analyses. Conserved protein motifs of MtPLATZ proteins were identified using the MEME Suite 5.5.9 (http://meme-suite.org/, accessed on 10 February 2026). The analysis was performed with the following parameters: a maximum of ten motifs per protein and a motif width ranging from 6 to 100 amino acids.

### 2.6. Expression Pattern of MtPLATZ Genes in Various Tissues/Organs

RNA-seq datasets encompassing diverse *M. truncatula* tissues were retrieved from the MtExpress database (https://medicago.toulouse.inrae.fr/MtExpress, accessed on 10 February 2026) [42]. The tissues included in the RNA-seq datasets were root, nodule, shoot, stem, leaf, flower, and developing embryonic tissues (embryo, endosperm, and seed coat). The expression levels of each *MtPLATZ* gene were quantified as Transcripts Per Million (TPM) values, which were normalized using the Trimmed Mean of M-values (TMM) method to correct for differences in library sizes among different RNA-seq samples. To visualize the tissue-specific expression patterns of *MtPLATZ* genes, a heatmap was generated using TBtools software. This heatmap was constructed based on the log_2_-transformed normalized expression values (TMM + 1), which facilitated the clear visualization and comparison of transcriptional dynamics of *MtPLATZ* genes across various organs and developmental stages.

### 2.7. Subcellular Localization Assay of MtPLATZ Proteins

To determine the subcellular localization of *MtPLATZ2*, *6*, *9*, *10*, *11*, *14*, *15*, and *16*, transgenic *Nicotiana benthamiana* plants stably expressing mCherry-NLS (a red fluorescent protein fused with a nuclear localization signal) were used [43]. Seeds were sown in a mixed substrate and grown in a growth chamber at 24–26 °C under a 16-h light/8-h dark photoperiod. Healthy 4- to 5-week-old plants were subjected to *Agrobacterium*-mediated transient transformation. Total RNA extracted from *M. truncatula* leaves was used to amplify each target CDS (without stop codon), which was cloned into pBin-GFP to produce *MtPLATZ*-GFP fusions driven by the CaMV 35S promoter. The resulting constructs were transformed into *A. tumefaciens* GV3101 via freeze–thaw method. Positive colonies were selected on YEB with kanamycin (50 mg/L) and rifampicin (50 mg/L), cultured overnight at 28 °C, harvested, and resuspended to OD_600_ 0.8–1.0 in infiltration buffer. After 2 h at room temperature, the suspension was infiltrated into leaves using a needleless syringe. Plants were kept in darkness for 24 h followed by 24 h of light. Leaf discs were examined under a Zeiss LSM880 confocal microscope (Carl Zeiss Microscopy GmbH, Oberkochen, Germany) for subcellular localization.

### 2.8. PLATZ Growth and Stress Treatments of M. truncatula Seedlings

Seeds of *M. truncatula* (accession Jemalong A17) were germinated and used for stress treatments. Seedlings were grown under controlled conditions (16 h light/8 h dark, 25 °C/20 °C day/night, 60% RH, light intensity: 150 μmol·m^−2^·s^−1^). To break dormancy, seed coats were lightly scored opposite the hilum, sterilized in 75% ethanol for 30 s, rinsed with sterile water, and germinated on moist filter paper at 25 °C. When roots reached ~2 cm, uniform seedlings were transplanted into pots and placed in a ventilated, shaded area.

To investigate responses to abiotic stress, leaf samples were collected from 4-week-old *M. truncatula* seedlings for stress experiments (including PEG-induced osmotic stress, acute NaCl shock, acute heat shock, and acute cold shock). Prior to treatment, the roots were rinsed with clean water, and the seedlings were acclimated for 1 day in Hoagland’s nutrient solution via hydroponics. Each independent hydroponic unit contained 250 mL of Hoagland’s nutrient solution. Continuous aeration was maintained throughout the culture period. Subsequently, abiotic stress was applied to the *M. truncatula* plants: 15% PEG6000 (*w*/*v*) was added to Hoagland’s medium to simulate water deficit (PEG-induced osmotic stress). Acute NaCl shock was induced by adding 300 mM NaCl to Hoagland’s medium. Acute cold and heat shock were induced by transferring the seedlings to growth chambers set at 4 °C and 42 °C, respectively. Both temperature treatments were conducted in identical growth chambers, with only the temperature setting differing between them; all other parameters were maintained strictly consistent. The pH of all stress treatment solutions was 5.8 at the start of treatment. Following stress treatment, leaves were collected at 0 (pretreatment baseline), 2, 4, 6, and 12 h. Samples were immediately immersed in liquid nitrogen for rapid freezing and subsequently stored at −80 °C for subsequent RNA extraction. Each sample was collected from 5 independent plants, and each experiment included three biological replicates.

### 2.9. RNA Extraction, cDNA Synthesis, and qRT-PCR Analysis

To analyze the expression of the *PLATZ* gene in *M. truncatula*, we used the SnapGene (version 8.2.2) software to design qRT-PCR primers for members of the *MtPLATZ* family. Total RNA was extracted using the SPARKeasy Plant RNA Kit (AC0305, Sparkjade Biotechnology Co., Ltd., Jinan, China), and RNA quality was assessed via 1% agarose gel electrophoresis [44]. cDNA was synthesized via reverse transcription using the SPARKscript II All-in-one RT SuperMixfor qPCR (AG0305, Sparkjade Biotechnology Co., Ltd., Jinan, China) and stored at −20 °C for later use [45]. Following the manufacturer’s recommendations, RNA samples were eluted with RNase-free ddH_2_O to remove DNA. qRT-PCR was performed using the 2xSYBR Green qPCR Mix (AH0104, Sparkjade Biotechnology Co., Ltd., Jinan, China) on a LightCycler^®^ 480 instrument (Roche Diagnostics, Basel, Switzerland). PCR parameters: 30 s pre-denaturation at 95 °C, 5 s denaturation at 95 °C, 30 s annealing/extension at 60 °C, for a total of 40 cycles; followed by melting curve analysis [46]. *Medtr3g095530* (*MtActin*) was used as the standard internal control gene [47], and the 2^−ΔΔCt^ method was employed to analyze the relative expression of each gene under different treatments [48]. Data analysis was performed using GraphPad Prism 8.0 software (https://www.graphpad.com, accessed on 17 May 2026). Results are expressed as the mean ± standard error of the mean (SEM) from three biological replicates. Comparisons between groups were performed using *t*-tests, with significance levels set at *p* < 0.05 (*), *p* < 0.01 (**), *p* < 0.001 (***) and *p* < 0.0001 (****).

## 3. Results

### 3.1. Identification of PLATZ Genes in M. truncatula

**Figure 1:**
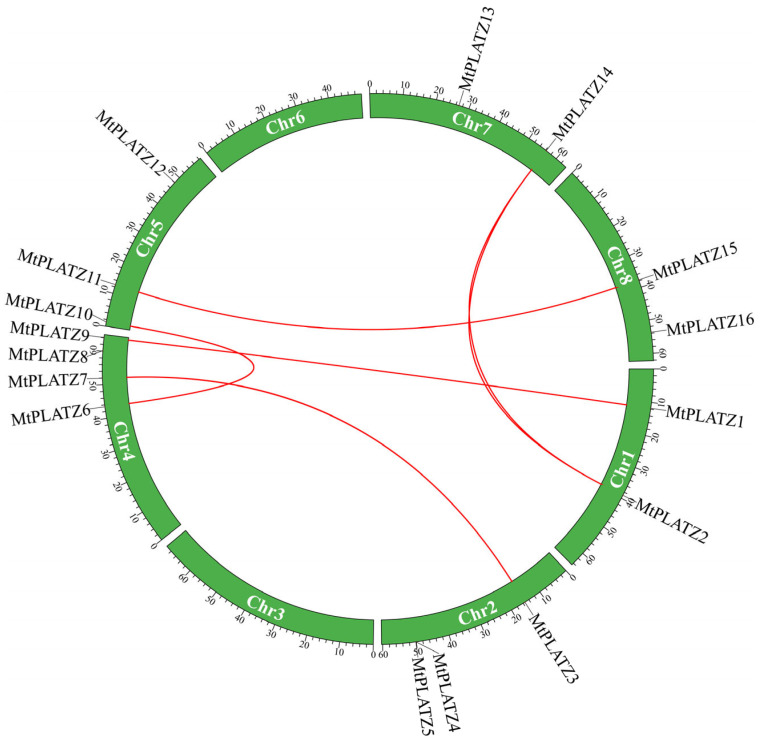
Chromosomal location and the syntenic relationships of *MtPLATZ* genes. The green outer ring corresponds to the eight chromosomes (Chr1 to Chr8) of *M. truncatula*, with the scales on the circle denoting the physical positions along each chromosome. Red curves indicate that genes generate homologous copies on different chromosomes via segmental duplication events.

A genome-wide screening approach combining HMMER and BLASTP algorithms led to the identification of 16 *MtPLATZ* genes in the *M. truncatula* genome. These genes were named *MtPLATZ1* through *MtPLATZ16* based on their sequential distribution across the chromosomes (Figure 1; Table 1). Protein sequence analysis demonstrated that 14 out of the 16 identified MtPLATZ members possess a highly conserved PLATZ domain, which harbors the consensus motifs M1 (spanning 33–38 amino acids) and M2 (19–21 amino acids), as well as an internal segment of 54–64 amino acid residues (Supplementary Table S1) [49]. Notably, *MtPLATZ8* contains a truncated M1 motif (13 amino acids) at its N-terminal region, whereas *MtPLATZ9* exhibits an extended internal sequence (76 amino acids) located between the M1 and M2 motifs.

The genomic DNA lengths of these *MtPLATZ* genes varied significantly, ranging from 1120 bp (*MtPLATZ5*) to 8832 bp (*MtPLATZ3*). In terms of protein size, *MtPLATZ8* and *MtPLATZ3* represented the shortest and longest members, with 154 and 258 amino acid residues, respectively. Physicochemical property analysis revealed that the molecular weights of MtPLATZ proteins ranged from 17.34 to 29.41 kDa, and their theoretical isoelectric points (pI) fell between 7.56 and 9.50, indicating that all MtPLATZ proteins are alkaline. All identified proteins were predicted to be unstable, with instability index values ranging from 40.44 to 67.36. The aliphatic index of these proteins varied from 45.40 to 77.33, suggesting relatively low thermal stability [50]. Additionally, the GRAVY scores of MtPLATZ proteins ranged from −1.01 to −0.26, which is indicative of their hydrophilic characteristics. Subcellular localization predictions showed that all MtPLATZ proteins are targeted to the nucleus (Table 1), which is consistent with their proposed role as transcription factors.

### 3.2. Analysis of Chromosomal Distribution and Synteny of MtPLATZ Genes

The chromosomal positions of the 16 *MtPLATZ* genes were mapped onto the chromosomes of *M. truncatula*, and the results showed an asymmetric distribution across six out of the eight chromosomes (Figure 1). Specifically, chromosome 4 contained the largest number of *MtPLATZ* genes (four), followed by chromosomes 2 and 5, each carrying three genes. Chromosomes 1, 7, and 8 each harbored two *MtPLATZ* genes, whereas no *MtPLATZ* gene was detected on chromosomes 3 and 6. According to well-accepted standards, gene pairs situated on the same chromosome with an intergenic distance of less than 200 kb are defined as tandem duplicates, while those located on different chromosomes are classified as segmental duplicates [51,52]. To explore the duplication events occurring within the *MtPLATZ* gene family, a collinearity analysis was conducted. This analysis identified five segmentally duplicated gene pairs and one tandemly duplicated gene pair (*MtPLATZ4* and *MtPLATZ5*) (Figure 1). Notably, the *K*a/*K*s ratios of all five segmental duplication pairs were substantially lower than 1, ranging from 0.12 to 0.35. This finding suggests that these segmentally duplicated *MtPLATZ* genes have experienced strong purifying selection, a process that maintains gene function by removing harmful mutations during the course of evolution (Supplementary Table S2).

### 3.3. Phylogenetic Analysis and Classification of MtPLATZ Genes

**Figure 2:**
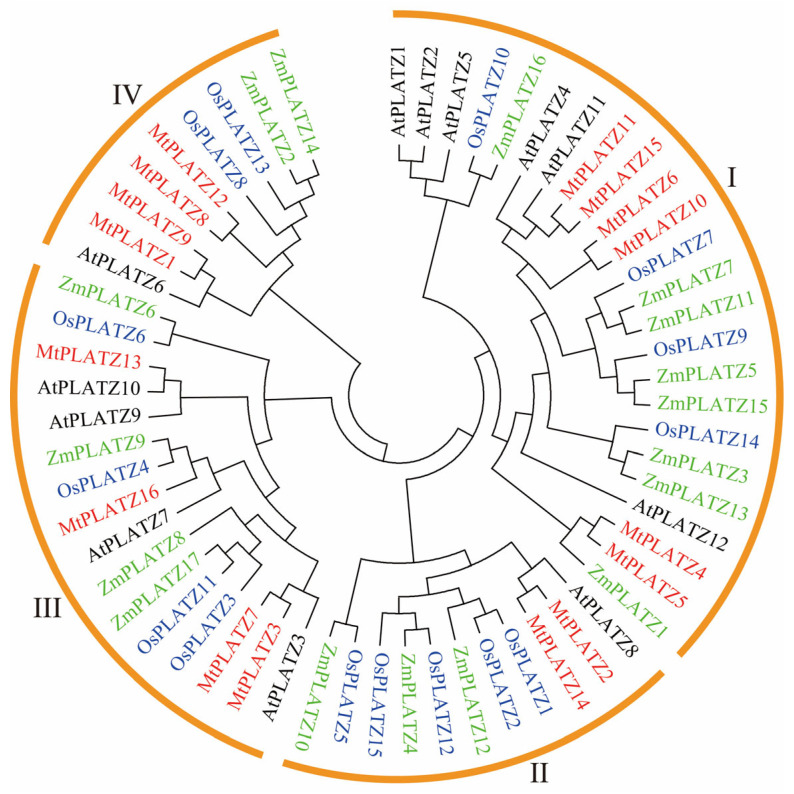
Phylogenetic analysis of PLATZ proteins from four plant species. The protein sequences of 12 AtPLATZs, 15 OsPLATZs, 16 MtPLATZs, and 17 ZmPLATZs were aligned using Clustal X software, and a neighbor-joining phylogenetic tree with 1000 bootstrap replicates was constructed using the MEGA 11.0 software.

To clarify the evolutionary relationships among PLATZ proteins, a phylogenetic analysis was performed using 60 PLATZ protein sequences derived from four angiosperm species, including 12 from *Arabidopsis*, 15 from rice, 16 from *M. truncatula*, and 17 from maize. Employing the neighbor-joining method, the constructed phylogenetic tree grouped all PLATZ proteins into four distinct clades (designated as Groups I–IV), with an asymmetric distribution of members across these clades (Figure 2). Despite the significant variation in the total number of PLATZ proteins among the four studied species, each phylogenetic group contained representatives from all four species. This observation implies both evolutionary conservation and potential functional divergence of the *PLATZ* gene family across angiosperms. Among the four groups, Group I was the most populous, encompassing 24 PLATZ proteins, followed by Group III (16 proteins), Group II (11 proteins), and Group IV (9 proteins). With respect to *M. truncatula*, its 16 MtPLATZ proteins were unevenly distributed across the four clades: six members clustered in Group I, four each in Groups III and IV, and only two in Group II. This distribution pattern suggests a biased evolutionary expansion or selective retention of specific MtPLATZ lineages during the evolutionary process of *M. truncatula*.

### 3.4. Gene Structure and Conserved Motif Distribution of MtPLATZs

**Figure 3:**
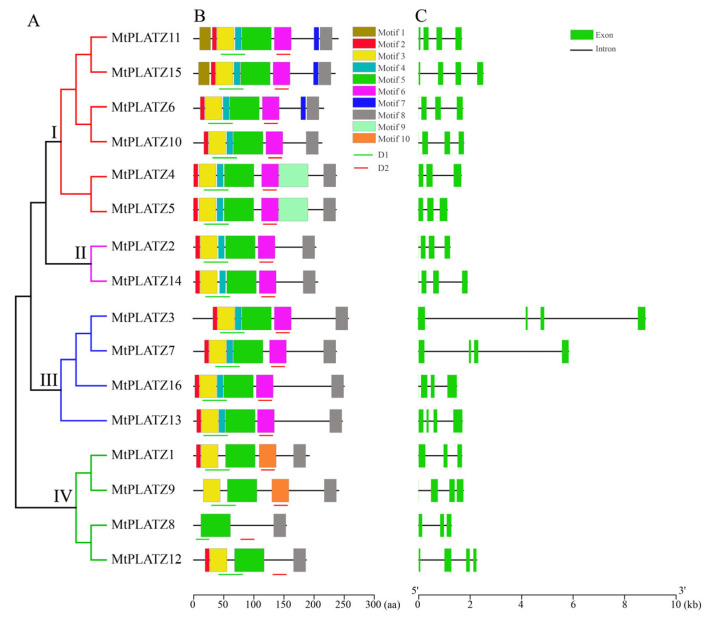
Schematic diagrams of *MtPLATZ* genes and motif composition of MtPLATZ proteins. (**A**) A neighbor-joining tree was constructed from the complete sequences of the 16 MtPLATZ proteins using MEGA11.0 software. (**B**) Distribution map of conserved motifs in MtPLATZ proteins. The ten putative motifs are represented by different colored boxes. The length of the exon/intron junctions and the amino acid sequences are inferred from the ruler at the bottom. (**C**) Structural analysis of the exon/intron distribution of the *MtPLATZ* genes, where the green boxes and black lines indicate the exons and introns, respectively.

Gene structure analysis demonstrated that 12 out of the 16 *MtPLATZ* genes contain four exons, whereas *MtPLATZ1*, *4*, *5*, and *8* are exceptional, each harboring three exons (Figure 3). Specifically, *MtPLATZ2*, *6*, *9*, *10*, *14*, and *16* possessed an extremely short first exon consisting of only the “ATG” start codon, while the first exon of the other *MtPLATZ* genes was relatively longer. Notably, the first and third introns of *MtPLATZ3* and *MtPLATZ7* were significantly longer than those of the other *MtPLATZ* genes, which contributed to the longer genomic lengths of these two genes. To explore the potential functional diversification of MtPLATZ proteins, a conserved motif analysis was performed, leading to the identification of ten conserved motifs with lengths ranging from 8 to 49 amino acids (Figure 3; Supplementary Figure S1). Motifs 2, 3, 4, 5, 6, and 8 were found to be universally present in all members of Groups I, II, and III. The conserved domain D1 corresponded to motifs 3, 4, and a small segment of motif 5, while domain D2 was associated with motif 6. Motifs 1 and 2 were specific to MtPLATZ11 and MtPLATZ15, respectively; motif 7 was exclusively detected in MtPLATZ6, 11, and 15; and motif 9 was unique to MtPLATZ4 and MtPLATZ5. Members of Group IV exhibited a distinct motif distribution pattern compared to those in other groups: all Group IV members lacked motifs 4 and 6, and MtPLATZ8 was deficient in motifs 1, 2, 3, and 4, which indicates that a truncation event occurred at its N-terminal region. Furthermore, most MtPLATZ proteins within the same phylogenetic group shared similar exon-intron organizations and conserved motif compositions, suggesting functional conservation among phylogenetically related members. In addition, the existence of group-specific motifs might imply potential subfunctionalization or neofunctionalization of *MtPLATZ* genes during the evolutionary process. These structural characteristics are well consistent with the phylogenetic classification results, which not only enhances the reliability of the group division but also provides valuable insights into the evolutionary dynamics of the *MtPLATZ* gene family.

### 3.5. Distribution of Cis-Acting Elements in MtPLATZ Gene Promoters

**Figure 4:**
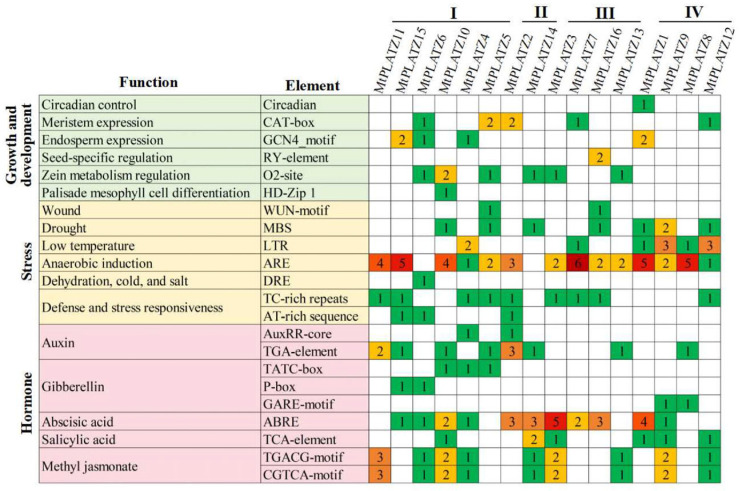
Prediction of *cis*-acting elements in *MtPLATZ* promoters. Colors and numbers of the grid indicate the number of different *cis*-acting elements in *MtPLATZ* genes. The numbers 1 (green), 2 (yellow), 3 (light orange), 4 (orange-red), 5 (red) and 6 (dark red) in the heatmap represent the copy number of each *cis*-acting element identified in the promoter sequences of *MtPLATZ* genes.

To explore the regulatory signals that modulate the expression of *MtPLATZ* genes, a 2.0 kb sequence upstream of the start codon of each *MtPLATZ* gene was extracted and submitted to the PlantCARE online server for *cis*-acting element prediction. A total of 22 distinct *cis*-acting elements were identified, which were classified into three categories: stress response, growth and development, and hormone response (Figure 4). Among the stress-responsive *cis*-acting elements, the anaerobic induction element was the most abundant, with 44 occurrences in total. This was followed by defense and stress responsiveness elements (12), cold-responsive elements (11), drought-responsive elements (8), wound-responsive elements (2), and one each of dehydration-, low-temperature-, salt stress-responsive elements and anoxic-inducible elements. Notably, every *MtPLATZ* promoter contained at least one type of stress-responsive element, and three promoters (*MtPLATZ5*, *12*, and *16*) harbored four different types of stress-responsive *cis*-acting elements. With respect to *cis*-acting elements associated with growth and development, meristem expression elements, zein metabolism regulation elements, and growth elements were the most prevalent, each with 7 occurrences. This was followed by seed-specific regulation elements (2) and a single circadian control element. *MtPLATZ6* contained three distinct types of *cis*-acting elements related to growth and development. The promoters of *MtPLATZ* genes were found to contain five categories of hormone-responsive *cis*-acting elements. Methyl jasmonate-responsive elements were the most abundant (28), followed by abscisic acid-responsive elements (26), auxin-responsive elements (13), and gibberellin-responsive elements (7). Importantly, *MtPLATZ10* was the only gene whose promoter contained all five types of hormone-responsive *cis*-acting elements.

### 3.6. Expression Profiles of MtPLATZ Genes in Different Organs and Developing Seed Tissues

**Figure 5:**
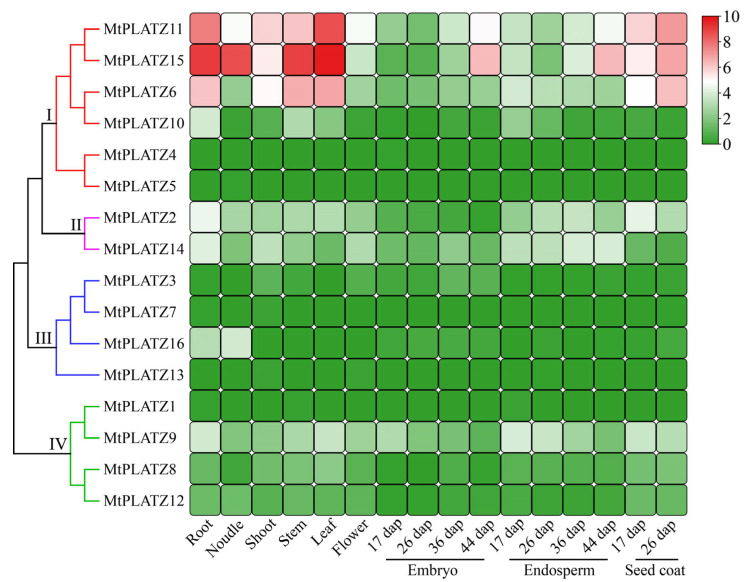
Expression profiles of *MtPLATZ* genes in different organs and developing seed tissues. The color scale from green to red indicates the increased expression levels of the genes.

To elucidate the expression patterns of *MtPLATZ* genes, the transcript abundance of these genes across diverse tissues and organs of *M. truncatula*—including roots, nodules, shoots, stems, leaves, flowers, developing embryos, endosperms, and seed coats—was analyzed using the MtExpress database. The gene expression values were normalized by employing the trimmed mean of M values (TMM) method [53]. For the classification of expression levels, genes with TMM values lower than 1.00 were defined as non-expressed, while those with TMM values ranging from 1.00 to 5.00, 5.00 to 20.00, and above 20.00 were categorized as lowly, moderately, and highly expressed, respectively. As illustrated in Figure 5 and Supplementary Table S3, the *MtPLATZ* genes exhibited distinct tissue-specific expression profiles. Specifically, *MtPLATZ1*, *3*, *4*, *7*, and *13* showed no detectable expression in any of the examined tissues, whereas the remaining 11 *MtPLATZ* genes were expressed in at least one tissue type. Among the expressed genes, *MtPLATZ5*, *8*, and *12* displayed extremely low expression levels (TMM < 4) and were only detected in a limited number of tissues. Two genes showed tissue-specific expression patterns: *MtPLATZ10* was exclusively expressed in roots, stems, leaves, and endosperms at 17/26 dap, while *MtPLATZ16* was moderately expressed solely in roots and nodules. The other six *MtPLATZ* genes were expressed in nearly all the tested tissues. Notably, *MtPLATZ6*, *11*, and *15* exhibited moderate to high expression levels in most organs, developing embryos, and seed coats, suggesting that these genes may be involved in fundamental growth and developmental processes of *M. truncatula*. In contrast, *MtPLATZ2*, *9*, and *14* showed relatively lower expression levels compared to *MtPLATZ6*, *11*, and *15* across the examined tissues. These results show that *MtPLATZ* genes are likely to participate broadly in the growth and development of *M. truncatula* and serve critical functions.

### 3.7. Expression of MtPLATZ Genes in Response to Abiotic Stress

**Figure 6:**
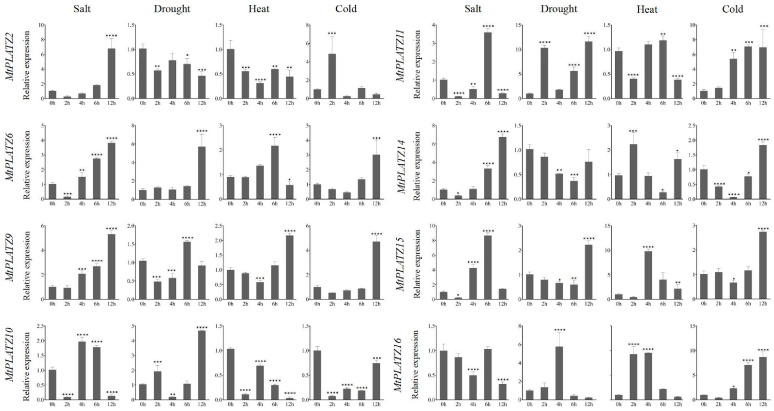
Relative expression levels of *MtPLATZ* genes under heat, cold, salt, and PEG stress in *M. truncatula*. qRT-PCR was used to examine the expression levels of eight leaf-expressed *MtPLATZ* genes, with significance indicated as *p* < 0.05 (*), *p* < 0.01 (**), *p* < 0.001 (***), and *p* < 0.0001 (****).

To investigate the potential functions of *MtPLATZ* genes in the stress responses of *M. truncatula*, we examined the expression levels of eight leaf-expressed *MtPLATZ* genes under PEG-induced osmotic stress, acute NaCl shock, cold (4 °C), and heat (42 °C) treatment using qRT-PCR (Figure 6; Supplementary Table S4). The results revealed that these genes exhibited distinct temporal expression patterns following treatment initiation. Under acute NaCl shock, *MtPLATZ2*, *MtPLATZ6*, *MtPLATZ9*, and *MtPLATZ14* showed elevated transcript levels, with *MtPLATZ2* and *MtPLATZ14* showing the highest induction (approximately 7- to 8-fold relative to 0 h), whereas *MtPLATZ16* did not change significantly throughout the time course. Under PEG-induced osmotic stress, most *MtPLATZ* genes were initially suppressed and then recovered; however, *MtPLATZ6* and *MtPLATZ10* exhibited a distinct expression peak at 12 h (approximately 5- to 6-fold of the 0 h). Notably, under acute heat shock, *MtPLATZ14* and *MtPLATZ16* were rapidly induced within 2 h, with expression levels increasing to 2.3-fold and 8-fold of the 0-h baseline, respectively; *MtPLATZ15* increased to 8-fold of the 0 h within 4 h of heat treatment. In contrast, the expression of *MtPLATZ2* and *MtPLATZ10* was significantly lower at several time points under heat stress. These results suggest possible functional divergence within this gene family in response to high temperature. Furthermore, acute cold shock induced only *MtPLATZ11* and *MtPLATZ16*, with no significant changes observed in the other members, suggesting a relatively limited role for the *MtPLATZ* family in low-temperature responses. Taken together, this preliminary expression profiling survey reveals that *MtPLATZ* genes exhibit diverse temporal expression patterns under different treatment conditions, implying their potential involvement in the adaptive responses of *M. truncatula* to environmental signals.

### 3.8. Subcellular Localization of Expressed MtPLATZ Genes

**Figure 7:**
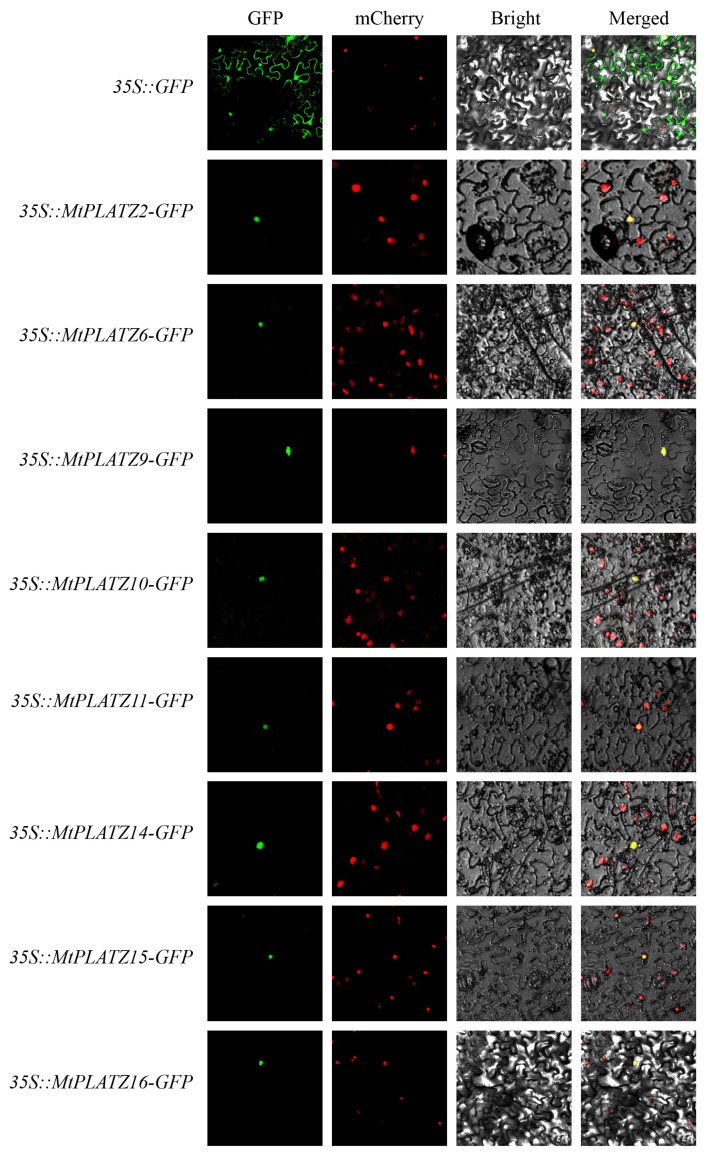
Subcellular localization of *MtPLATZs* in *N. benthamiana* leaves. *pBin-GFP* was used as a control, and mCherry fusion protein was used as a nuclear localization marker. GFP, green fluorescence of fusion proteins; mCherry, monomeric cherry fluorescent protein; Merged, merged microscopic images.

To explore the transcriptional characteristics of *MtPLATZ* genes, eight expressed members (*MtPLATZ2*, *6*, *9*, *10*, *11*, *14*, *15*, and *16*) were selected for a subcellular localization assay (Figure 7). The CDSs of these *MtPLATZ* genes, excluding the stop codon, were fused to the N-terminus of green fluorescent protein (GFP), with the fusion constructs under the regulation of the CaMV 35S promoter. *Agrobacterium* cultures harboring the recombinant constructs were used to infiltrate the epidermal cells of tobacco leaves, with the *35S::GFP* empty vector serving as the negative control. The subcellular localization of GFP signals in *N. benthamiana* leaves was observed and recorded using confocal microscopy at 48 h post-transformation. As presented in Figure 7, the GFP signal of the control group was uniformly distributed across the entire cell. In contrast, all MtPLATZ-GFP fusion proteins were specifically localized in the nucleus. These findings confirm that the selected MtPLATZ proteins are nucleus-localized proteins, which is consistent with their putative function as transcription factors.

## 4. Discussion

The cell nucleus is one of the most information-dense regulatory centers within a cell, continuously receiving various internal and external signals and dynamically adjusting its structure and gene expression activity accordingly [54,55]. PLATZ proteins are a class of plant-specific zinc-dependent DNA-binding proteins. They regulate the expression of target genes by binding to specific DNA sequences in the nucleus and play a crucial role in plant growth, development, and responses to abiotic stress [23]. Previous studies have shown that members of the *PLATZ* gene family are extensively involved in developmental processes such as leaf development and senescence [14], cell proliferation [27], and grain morphogenesis [15], and also exhibit important functions in responses to abiotic stresses including drought [29], salinity [25], and extreme temperatures [56]. To date, the *PLATZ* family has been systematically characterized in model plants such as *Arabidopsis* [17], rice [16], and tomato [56]. It has also been identified in important crops including wheat [22], maize [17], and upland cotton [25], as well as in other plant species such as pepper [12], ginkgo [13], apple [57], and Chinese cabbage [58]. These studies have laid a foundation for elucidating the structural characteristics, evolutionary patterns, and functional diversity of the *PLATZ* gene family. The unique value of *M. truncatula* as a well-established model legume resides in its dual nature: it offers both the experimental tractability of a model system (small genome, efficient transformation, and abundant genomic resources) and the agronomic relevance of a major legume crop. However, a genome-wide systematic identification and analysis of the *PLATZ* genes has not yet been performed in the legume model plant *M. truncatula*.

In this study, a total of 16 *MtPLATZ* genes were systematically identified in the *M. truncatula* genome through genome-wide analysis (Figure 1; Table 1), providing important insights into their potential roles in plant growth, development, and abiotic stress tolerance. Comparative analysis with previously reported *PLATZ* members in other species revealed that *Arabidopsis* contains 12 members [14] and rice contains 15 members [16]. In contrast, soybean contains as many as 29 members [21], attributed to multiple whole-genome duplication events. The number of *PLATZ* genes in soybean is approximately 2.23-fold that of *Arabidopsis*, 1.93-fold that of rice, and 1.81-fold that of *M. truncatula*. Thus, the *PLATZ* gene number in *M. truncatula* is comparable to that in *Arabidopsis* and rice, suggesting that these species have roughly similar genome sizes, which are smaller than that of soybean [21]. Analysis of the protein sequences encoded by these genes revealed that 14 *MtPLATZ* members contain a highly conserved PLATZ domain, located between C-x_2_-H-x_11_-C-x_2_-C-x_(4–5)_-C-x_2_-C-x_(3–7)_-H-x_2_-H and C-x_2_-C-x_(10–11)_-C-x_3_-C. This domain is proposed to function as a zinc-binding motif rich in histidine and cysteine, thereby constituting the core feature distinguishing the PLATZ family from other transcription factors [7]. On this basis, further analysis demonstrated significant variation in *MtPLATZ* gene lengths (1120–8832 bp) (Table 1). It is therefore suggested that this family may have undergone varying degrees of genetic structural rearrangement during evolution [16]. Subcellular localization prediction revealed that MtPLATZ proteins are largely localized to the nucleus and possess high isoelectric points, consistent with their properties as DNA-binding proteins (Table 1) [16,17,59]. To further corroborate this, we selected eight representative members with relatively high expression levels and confirmed their nuclear localization via subcellular localization assays (Figure 7) [16]. All members were computationally predicted to be unstable proteins based on instability index analysis—a feature also observed in the soybean *PLATZ* gene family. This has been suggested to be potentially associated with their transient expression patterns during seed development and stress responses, although this relationship requires experimental verification [21]. Gene duplication has been recognized as one of the most fundamental drivers of evolutionary innovation, as it contributes to increased biological diversity and adaptability [60]. This study demonstrated that five *MtPLATZ* gene pairs underwent segmental duplication, while one pair underwent tandem duplication. Furthermore, the *K*a/*K*s ratios of all five duplicated gene pairs were less than 1 (Figure 1; Supplementary Table S2), suggesting that purifying selection has significantly influenced the evolution of the *MtPLATZ* family. Previous studies have shown that both segmental and tandem duplications are present in the *PLATZ* gene families of tomato and apple [56,57]. It is therefore proposed that segmental duplication represents the primary and most widespread mechanism driving the expansion of the *PLATZ* gene family in higher plants. Moreover, motif distribution analysis elucidated that most MtPLATZ share a set of core motifs (e.g., Motif 1–Motif 10), which potentially play critical roles in MtPLATZ protein function and have been subject to strong purifying selection during evolution (Figure 3; Supplementary Figure S1) [61,62].

Given the close link between evolutionary clustering and functional differentiation, we performed phylogenetic analysis using 60 PLATZ proteins derived from *Arabidopsis*, rice, maize, and *M. truncatula* (Figure 2). These proteins were clustered into four subfamilies, consistent with previous findings [36,63,64]. This indicates that the core structural features of the PLATZ family were possibly established before the divergence of monocots and dicots [65]. Subsequent analyses of gene structure and conserved motifs demonstrated that *MtPLATZ* genes within the same subgroup share highly similar conserved features, further validating the reliability of their phylogenetic grouping. Notably, a certain degree of evolutionary conservation was observed between *MtPLATZ* genes and their counterparts in *Arabidopsis*, rice, and maize, whereas species-specific characteristics were also detected. Therefore, Group I, which is present in all four species and contains the most members, may represent a more conserved and functionally fundamental subfamily [66,67]. Whole-genome duplication (WGD) and segmental duplication events have been shown to jointly drive the expansion of the *PLATZ* gene family [68]. In contrast, Group II comprised only three dicotyledon members, whereas monocotyledons accounted for eight. A distinct enrichment of monocotyledons was observed in this group, suggesting that this subfamily may have undergone specific expansion in monocotyledons, potentially associated with physiological or developmental processes unique to this lineage [69]. Furthermore, all four species in Group III possessed exactly the same number of genes (four each), demonstrating strict conservation of gene numbers across species—a phenomenon considered relatively rare in previous *PLATZ* family studies. Subfamily I and Subfamily III contained equal numbers of monocot and dicot members, indicating that these two clades have experienced strong purifying selection, and their sequences and functions have remained stable after the divergence of higher plants. Notably, inter-species variation in *PLATZ* gene numbers was minimal across all groups. Finally, the number of *PLATZ* genes in Group IV was significantly higher than that in other species. This group exhibited lineage-specific expansion in legumes, suggesting a possible functional divergence of this subfamily in legumes; however, whether such functional specialization involves symbiotic nitrogen fixation or nodule development—biological processes unique to legumes—remains to be tested by future functional experiments [70]. Moreover, *MtPLATZ* genes from different lineages may be involved in distinct biological processes, including growth and development, seed development, hormone response, and stress adaptation [18,23].

Structural conservation and divergence are key to resolving evolutionary dynamics and functional differentiation in gene families [71]. In this study, most *MtPLATZ* genes were found to harbor a highly conserved four-exon structure, indicating that the PLATZ family has experienced strong evolutionary constraints in the *M. truncatula* genome (Figure 3). The retention of core coding regions during long-term evolution guarantees the conserved DNA-binding function of this family [43]. Notably, several family members, including *MtPLATZ1*, *MtPLATZ4*, *MtPLATZ5* and *MtPLATZ8*, possessed fewer exons and only retained three exons in their gene structures. Previous studies have demonstrated that exon loss is a prevalent evolutionary event accompanying the expansion of plant gene families, which is closely associated with functional specialization, altered expression patterns and adaptive evolution under stress [72]. Changes in exon-intron architecture can directly affect transcription efficiency, mRNA splicing and the final protein spatial structure, thereby driving functional divergence among homologous genes [73]. Accordingly, the structural variations of several *MtPLATZ* genes in this study not only reflect the structural diversity within the family, but also suggest that these members may have evolved distinct biological functions compared with canonical *PLATZ* genes.

Promoter *cis*-acting elements are essential functional regulators that govern the spatiotemporal expression of genes and mediate plant responses to internal and external stimuli. Their distribution patterns may provide useful clues for understanding the functional divergence and regulatory properties of gene families [74]. In this study, characterization of promoter sequences across the *MtPLATZ* family identified abundant *cis*-elements associated with stress responses, hormone signaling, as well as plant growth and development (Figure 4). These findings suggest that *MtPLATZ* genes may be involved in the modulation of growth, developmental processes and stress adaptation in *M. truncatula*; however, this inference requires experimental validation. *Cis*-elements related to anaerobic induction, defense responses, low temperature and drought were highly enriched in *MtPLATZ* promoters. Notably, every member of this family harbored at least one type of stress-responsive element, implying that *MtPLATZ* genes might be broadly involved in diverse biotic and abiotic stress pathways [75]. Several genes, namely *MtPLATZ5*, *MtPLATZ12* and *MtPLATZ16*, contained multiple stress-related *cis*-elements. This evidence suggests that these genes may respond to a wide range of stress signals and potentially act as important components within the stress regulatory network of *M. truncatula* [23]. The distinct distribution of hormone-responsive *cis*-elements further indicates that the biological functions of the *MtPLATZ* family are modulated through hormone signaling cascades [76]. Methyl jasmonate- and abscisic acid-responsive elements were the most prevalent in *MtPLATZ* promoters, followed by auxin- and gibberellin-responsive elements. This distribution pattern suggests that the *MtPLATZ* family might rely on methyl jasmonate and abscisic acid signaling to regulate plant stress tolerance and growth [19], although this hypothesis awaits verification through molecular and genetic approaches. As core signaling hormones for plant stress defense, the high abundance of their corresponding *cis*-elements further supports the stress-resistance functions of *MtPLATZ* genes, rather than constituting direct proof. In addition, the promoter of *MtPLATZ10* was found to possess all five categories of hormone-responsive elements. This unique feature implies that *MtPLATZ10* may engage in extensive crosstalk among multiple hormone signals and execute a distinctive function in balancing plant growth and stress responses [77], but this possibility requires experimental validation.

Significant variation in the expression of *PLATZ* genes across different tissues was observed, suggesting that this gene family may have multiple functions. Public database analysis revealed the expression patterns of each *MtPLATZ* gene across six organs and different seed developmental stages. Among the 16 *MtPLATZ* genes, 11 were expressed in at least one tissue or organ, whereas the remaining five showed no expression in any of the samples examined (Figure 5; Supplementary Table S3). In contrast, *MtPLATZ6*, *MtPLATZ11*, and *MtPLATZ15* were expressed in most of the tested organs and tissues, indicating that they may possess broad-spectrum expression characteristics throughout the entire plant. These three genes were predominantly expressed in vegetative organs (roots, root nodules, stems, and leaves) but showed low expression in other tissues, suggesting a possible tissue-specific pattern. The remaining members displayed tissue- or organ-specific expression patterns. For example, *MtPLATZ2* was detected in roots and 17 dpa seeds, whereas *MtPLATZ9* was expressed in roots, leaves, the endosperm at 17 dpa after pollination, and 17 dpa seeds. Additionally, *MtPLATZ16* was specifically expressed in roots and root nodules. Collectively, these expression profiles revealed a clear functional divergence within the *MtPLATZ* gene family.

Downstream gene expression is thought to assist plants in responding to various environmental stresses—including salinity, drought, extreme temperatures, and pathogen infection—which have been extensively characterized across diverse species [23,78,79]. In many plant species, abiotic stresses such as drought, high salinity, or extreme temperatures upregulate *PLATZ* gene expression, and some *PLATZ* genes are believed to enhance plant tolerance to these stresses. For example, drought stress promotes the expression of *GhPLATZ01* and *GhPLATZ15*. Heterologous overexpression of these upland cotton genes in tomato significantly enhanced drought tolerance—an effect attributed to increased chlorophyll, relative water content, and proline levels; reduced malondialdehyde content and electrical conductivity; and upregulated expression of antioxidant enzyme genes, thereby alleviating ROS stress [26]. Tomato lines overexpressing *SlPLATZ17* also exhibited greater salt tolerance. Transcriptomic analysis suggested that this gene may act through interaction with POR1 to modulate glutathione, arginine, and proline metabolism [80]. Moreover, twenty *MsPLATZ* genes in alfalfa were found to respond simultaneously to salt, cold, and drought stress [56]. Wheat *TaPLATZ5* was shown to be induced by powdery mildew infection and to interact with the histone acetyltransferase *TaHAG1*, thereby activating *TaPAD4* expression through an additive effect and positively regulating powdery mildew resistance [81]. To explore the expression profiles and potential functions of *MtPLATZ* genes in response to abiotic stresses, eight genes with distinctly high expression levels were selected for qRT-PCR analysis in this study. Their relative transcript levels were measured at 0 (pretreatment baseline), 2, 4, 6, and 12 h under PEG-induced osmotic, NaCl, heat, and cold treatment conditions. The results showed that most of the eight genes were significantly induced or repressed under PEG-induced osmotic stress, acute NaCl shock, acute heat shock, and acute cold shock, suggesting their possible involvement in the abiotic stress response of *M. truncatula* seedlings (Figure 6). Notably, *MtPLATZ9* exhibited a steadily upregulated expression pattern under all four stresses tested—PEG-induced osmotic, heat, cold, and NaCl—making it the only gene in this study that consistently responded positively to all four types of abiotic stress, thus indicating its potential role as a broad-spectrum stress-responsive gene. *MtPLATZ6* displayed strong late upregulation at 12 h under PEG-induced osmotic, cold, and NaCl treatments, implying its possible participation in metabolic regulation or damage repair processes during the late phase of stress exposure. This expression pattern suggests that *MtPLATZ6* is possibly sensitive to prolonged stress and possesses a prominent late-acting stress resistance function [82]. The expression of *MtPLATZ2* was significantly suppressed under PEG-induced osmotic, heat, and cold treatments, but was rapidly upregulated at 12 h under NaCl stress, with an increase of approximately 7-fold relative to 0 h. Collectively, these results suggest that *MtPLATZ* family members cooperatively may mediate the adaptive responses of *M. truncatula* to multiple abiotic stresses through temporally and spatially differential expression patterns.

A limitation of the experimental design should be acknowledged: no mock-treated controls were included at each sampling time point during the stress treatment time course. Consequently, the observed expression changes under stress conditions might be partly confounded by circadian rhythms or environmental fluctuations. For instance, the endogenous circadian clock in *Arabidopsis* regulates the rhythmic oscillation of up to 30% of transcripts at different times of the day [83]. Nonetheless, by comparing the expression levels at each stress time point with the initial (0 h) baseline, we were able to preliminarily identify trends of stress-induced or stress-repressed expression, which is particularly informative for continuously upregulated or time-dependent expression patterns. In future studies, a more systematic experimental design will be adopted to obtain more robust evidence. This study provides valuable insights into the *MtPLATZ* gene family; however, additional genetic and physiological experiments are warranted to definitively characterize the stress-responsive functions of individual *MtPLATZ* members.

## 5. Conclusions

In this study, we identified a total of 16 members of the *MtPLATZ* gene family in *M. truncatula*, which are unevenly distributed across six chromosomes. Phylogenetic analysis revealed that these 16 genes are grouped into four clusters; members within the same subcluster share a unique evolutionary history and perform related biological functions. Notably, gene duplication events were observed within the *MtPLATZ* gene family, with five pairs of *MtPLATZ* genes exhibiting segmental duplications and one pair exhibiting tandem duplications. We also found that the anaerobic response element (ARE) is widely present in the *cis*-acting elements of *MtPLATZ* promoters, which may suggest that the *MtPLATZ* family primarily functions in stress responses. Furthermore, based on RNA-seq data and qRT-PCR analysis, the expression of these *MtPLATZ* genes exhibits diversity across different organs. Importantly, the expression levels of most *MtPLATZ* genes were altered in leaves under various abiotic stress conditions, including PEG-induced osmotic stress, acute NaCl shock, cold (4 °C), and heat (42 °C) treatment. The results of this study may serve as a valuable resource for future functional characterization of *MtPLATZ* genes and for the genetic improvement of stress tolerance in *M. truncatula*.

## Figures and Tables

**Table 1:** Physicochemical properties and subcellular localization prediction of *MtPLATZ* members.

Name	Gene ID	Chromosomal Location (Strand)	Genomic Length (bp)	Protein Length	Molecular Weight (kDa)	Isoelectric Point	Instability Index	Aliphatic Index	GRAVY	Subcellular Localization
*MtPLATZ1*	*MtA17chr1000000000g1316*	chr1:11750444-11752123(+)	1680	192	22.37	8.74	40.44	66.98	−0.86	Nucleus
*MtPLATZ2*	*MtA17chr1000000000g3531*	chr1:38991018-38992162(+)	1145	203	22.95	8.83	62.05	72.02	−0.43	Nucleus
*MtPLATZ3*	*MtA17chr2000000000g8376*	chr2:15389491-15398322(+)	8832	258	29.41	7.56	55.25	70.62	−0.47	Nucleus
*MtPLATZ4*	*MtA17chr2000000000g11348*	chr2:50104564-50106219(−)	1656	237	27.57	8.55	60.47	71.98	−0.72	Nucleus
*MtPLATZ5*	*MtA17chr2000000000g11352*	chr2:50110603-50111722(−)	1120	237	27.44	9.22	56.56	66.24	−0.80	Nucleus
*MtPLATZ6*	*MtA17chr4000000000g23973*	chr4:43420605-43422215(−)	1611	216	23.96	9.31	53.84	67.18	−0.54	Nucleus
*MtPLATZ7*	*MtA17chr4000000000g25051*	chr4:51963647-51969469(−)	5823	237	26.59	8.21	48.84	70.72	−0.42	Nucleus
*MtPLATZ8*	*MtA17chr4000000000g26038*	chr4:60157350-60158619(−)	1270	154	17.34	8.88	50.99	65.13	−0.66	Nucleus
*MtPLATZ9*	*MtA17chr4000000000g26522*	chr4:64111459-64112721(+)	1263	241	27.89	8.76	55.35	52.53	−0.96	Nucleus
*MtPLATZ10*	*MtA17chr5000000000g26797*	chr5:1259107-1260723(+)	1617	213	23.97	9.08	63.58	75.92	−0.49	Nucleus
*MtPLATZ11*	*MtA17chr5000000000g28169*	chr5:12701500-12703169(−)	1670	240	27.47	9.41	51.36	72.29	−0.66	Nucleus
*MtPLATZ12*	*MtA17chr5000000000g31352*	chr5:48055705-48057736(+)	2032	187	21.67	9.46	63.04	45.40	−1.01	Nucleus
*MtPLATZ13*	*MtA17chr7000000000g39610*	chr7:26883965-26885661(−)	1697	247	27.44	9.10	67.36	77.33	−0.26	Nucleus
*MtPLATZ14*	*MtA17chr7000000000g43012*	chr7:56241807-56243590(−)	1784	206	23.16	8.83	63.38	76.17	−0.39	Nucleus
*MtPLATZ15*	*MtA17chr8000000000g47466*	chr8:37709228-37711733(−)	2506	235	26.74	9.50	50.23	69.66	−0.60	Nucleus
*MtPLATZ16*	*MtA17chr8000000000g49218*	chr8:53674680-53676071(−)	1392	250	28.73	8.49	65.07	66.24	−0.46	Nucleus

## Data Availability

Please contact the corresponding author for any additional information.

## Supplementary Materials

The following supporting information can be downloaded at: https://www.mdpi.com/article/10.3390/biology15151318/s1, Figure S1: The sequences information of 10 conserved motifs identified in MtPLATZ proteins; Supplementary Table S1: Length of M1 and M2 domains, and their internal sequences in MtPLATZ proteins; Supplementary Table S2: The *K*a/*K*s ratios of the duplicated *MtPLATZ* gene pairs; Supplementary Table S3: TMM values of *MtPLATZ* genes in different organs and developing seed tissues of *M. truncatula*: Supplementary Table S4: The sequences of the primers used for the qRT-PCR analysis.
