# Supplementary material for: Genome-Wide Characterization of the PLATZ Gene Family in Medicago truncatula and Their Expression Profiles in Response to Abiotic Stress
Source: Biology (Basel). 2026 Aug 6;15(15):1318. doi: 10.3390/biology15151318 (PMC13466130; doi:10.3390/biology15151318)
Supplement: Supplementary file 1 [file biology-15-01318-s001.zip › biology-4441578-supplementary.pdf]

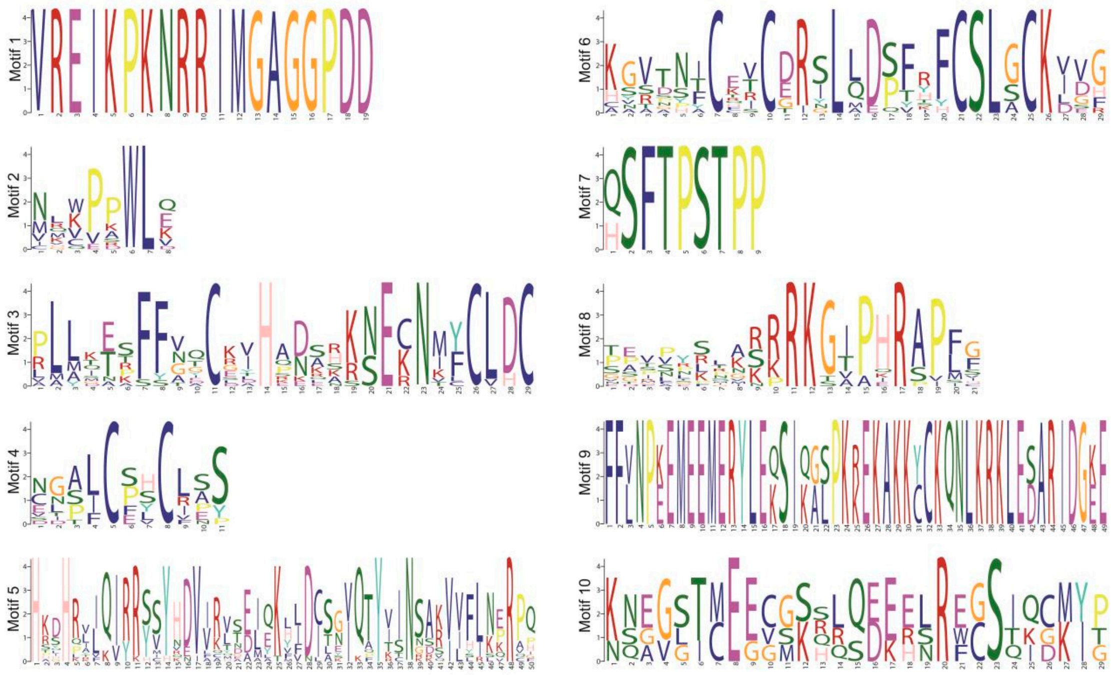

Motifs	Sequence	Width
Motif 1	VREIKPKNRRIMGAGGPDD	19
Motif 2	NLKPPWLE	8
Motif 3	PLLKESFFVQCKIHADSRKNECNMYCLDC	29
Motif 4	NGALCPHCLSS	11
Motif 5	HKDHRVJQIRRSSYHDVIRVSEJQKLJDCSGVQTYVINSAKVVFLNERPQ	50
Motif 6	KGVTNICEVCDRSLLDPFRFCSLGCKIVG	29
Motif 7	QSFTPSTPP	9
Motif 8	TEVPYSLARRRKGIPHRAPFG	21
Motif 9	FFLNPEEMEEMERYLEKSIKGLPKKEKAKKCCKQNLKRKLEDARIDGEE	49
Motif 10	KNEGSTCEECGKQLQDEELREGSIQCKYP	29

Figure S1 The sequences information of 10 conserved motifs identified in MtPLATZ proteins.

Supplementary Table S1 Length of M1 and M2 domains, and their internal sequences in MtPLATZ proteins

Group	Name	Length of M1	Length between M1 and M2	Length of M2
I	MtPLATZ11	35	56	19
I	MtPLATZ15	35	56	19
I	MtPLATZ6	35	56	19
I	MtPLATZ10	35	56	19
I	MtPLATZ4	36	64	19
I	MtPLATZ5	36	64	19
II	MtPLATZ2	36	56	19
II	MtPLATZ14	38	56	19
III	MtPLATZ3	34	56	19
III	MtPLATZ7	34	62	19
III	MtPLATZ16	34	56	19
III	MtPLATZ13	34	55	19
IV	MtPLATZ1	33	58	20
IV	MtPLATZ9	34	76	20
IV	MtPLATZ8	13	56	21
IV	MtPLATZ12	35	54	21

The following is the protein sequence of MtPLATZ, with the M1 and M2 sequence regions marked with single and double underscores, respectively.

>MtPLATZ1
MVKRSLPNGKEDWLDALLMDRFGNCKNHKEKFNEKNVFCLDCGISFCRYDKESHSLHR
KVQIYRYCYVDVAKYTDLLKYFDCSYIQTYISNNDKIVLIKPRPPKTSTSQKQAGGICEEC
GKQLQDKRNRFCSIKCKITIEELGINQDDHQNSEAESSISTDEPNESGERVNLRKRPRKSTPI
RSISSFVNDY

>MtPLATZ2
MDTMLVPKWLKPLLSTPFFNECRIHADAARSECNMFCLDCNVDAFCFYCRSSKHKDHQV
IQIRRSSYHDVVRVAEIQKMLDISGVQTYVINSARVLFLNERPQPKSGKAVPYICEICGRGL
LDQVRFCSLGCKLVGIKRNGNASFVLDANNNEVSTMEEGMSRQQEEELREGSQQGMYPA
TPPTPPSNARRRKGIPHRAPFGP

>MtPLATZ3
MVRDMMMGTSTYNSISSSSSRGDMNMDMDMDMMNMKPAWLERLMDETFFGDCGVHK
NRRKNEKNIFCLHCCLSICPHCLSSHTSHPLLQVRRYVYHNVIRLDDLEKLIDCSNIQPYTI
NSAKVIFLNQRPQSKSCKVTTNACFTCDRILQDPFHFCSLSCKVDFMVYEGQSLSNILHRF
DESDLGISQFEGLRMDGSEVIDEDNNHIGPSSSNYSITHQEEATSNKITKKNKSNRFLPGIVL
SLGSRRKGAPQRAPLS

>MtPLATZ4
MMCVPWLVNLLAITSFFATCEAHPNKSRNERNKFCLDCNENPLCESCIESRHKDHLVIQIR
RSSYNEVVKTTEIYKHVDLLGIQTYVINSSSVVYLNKRAHAQSKRFDLEKIGHSRDSFCKR
CDIYLMDSTYFCSLACKDGGFFLNPEEMEEMERYLEKSIKGLPKREKAKKYCKQNLKRK
LEDARIDGEEGEQDVEKEKEESVDDKESILPVNRSSASLRKPNSKRKGIAHRAPFF

>MtPLATZ5
MLCVPWLVNLLAITSFFTTCETHPKKSKNERNKFCLDCNENPLCESCIESRHRGHRVIQIRR
SSYNEVIKTTEIYKHVDILGIQTYVINSSSVVFLSKRAHAQPKKFNIGKTGHYRDPFCKRCD
RNLADPTYFCSLACKDGGFFVNPKEMEEMERYLEQSIQASPKKEKAKKCCKQNLKRKLE
SARIDGKEEEENVEKEREEGADDRESILPVNRSSASLRKPNSKRKGIAHRAPFF
>MtPLATZ6
MGGGGGNNEEENKWPPWLQPLLQTSFFVQCKVHSDSHKSECNMYCLDCNNGALCSVCL
ASHKQHRTIQIRRSSYHDVIRVSEIQKFLDIAEVQTYVINSAKIVFLNERPQPRPGKGVTNT
CQVCDRSLLDSFSFCSLGCKIVGTSKKLRKNKMLSQKDGSDGEESMNGISNGSGRNRSHS
FTPSTPPPTAVSYRTVKRRKGIPHRAPMGGGIIIEF
>MtPLATZ7
MRGSGSDPSSEAKGDMKVNQKPAWLERLMGETFFGGCGVHQNQRKNEKNILCLHCCLTI
CPHCLPSHPSHPLLQVRRYVYHDVVQLGDLEKLIDCSNIQAYTINGAKVIFLNERVQSRSC
KGTTTAANANSFCCTCDRILQDPFHFCSLSCKVDHMVYKGESLSSILVHRFHESHFSYSQF
EGLRVDSSEVIDEDNTQFVPTNTEEATSRSMNNKTNGFFLSLGSRRKGAPKRAPLI
>MtPLATZ8
MVCCQYCISSGVHRHHKILKIYRHIYKDVVALSAMEKYFDCSEVQTYKSNRKVVISLKPR
QFSEIASNAEDSCEVCNKKLNEPDLYRYCALSCKVEAVSKKSESEDPVSTTPLESSSGPSIQ
GPPQHQETQEGTSGPSLGKRKRKGIPHRSPFF
>MtPLATZ9
MVGKDQSPKERNRDWIRALMNSSSGYCDDHRDLRSNEKNTFCVDCAVRFCRHCKEAHSI
HRRFQIYRYSYQDVFRHSELQKHFDCSNIQTYISNKDRIVHLKPRPPIYKSKSGDQCPESKS
KESNLSARFKSGGSTCEECGKHLQDEHSRWCSIICKIGEPQSQGPNHHTERSLVDSFNQSG
RCIITPKTEAIDFTMSDNLNSEPESSISEAEPCGRVEVVNFRKRPRKTTPERPVFVFVS
>MtPLATZ10
MGDGEGVVGVRSLNEEEIKWPPWLQPLLQARFFVQCKVHADSNKSECNMYCLDCMNG
ALCSSCLASHKDHRAIQIRRSSYHDVIRVSEIQKFLDITGVQTYVINSAKIVFLNERPQPKPG
KGVTNICHVCERSLLDSFTYCSLGCKIVGTSKKFQKKLSERHRSEGEESYSNENDININKT
QSFTPSTPPPYKIAKRRKGVPHRAPIGGGLIY
>MtPLATZ11
MAIENQENTSVREIKPKNRRIMGAGGPDDDDNRWPPWLKPLLKEAFFVQCKIHADSHKS
ECNMYCLDCMNGPLCSLCLAYHKDHRYIQIRRSSYHDVIRVNEIQKHLDIAGVQTYIINSA
RVVFLNERPQPRPGKGVTNTCEVCERSLLDSFRFCSLGCKIVGTSKNFQKKKRHLTAMAS
DSEESYSSNNIHSRQKKIKVQSFTPSTPPPTSVNYRTAKRRKGIPHRAPLGGLDVDYYLL
>MtPLATZ12
MSSSSSRKMNKDEQVAQPEVHQPKWLQDFLGKKFFRACSAHSDRRNELNIYCINCKESA
CQYGLSSGFHHDHRILKIYKYMHRDVVCQTAMQTYINCSKIKQYKCNNRQVLHRLPRCG
STLDDTSSCSFGSRNSNGANSYQYCSIACKYKDMSRKSEDSIPTRESQGETSEPQKRKRKG
TPHRAPFF
>MtPLATZ13
MCSSPNVVPRWLQVMLTEKFYNACLIHQEEKKNEKNIYCLDCSISLCPHCVSPHASHRLL
QIRRYVYHDIIRMDDAAKLIDCSSIQFYRTNGAKVIFINQRPIPKKCSSSNICRTCTRSLQDP
YKFCSLSCKVSHLVKTFGSLSGYLVECNTMPPLSDSGLDDSLMTPDSVLEPSGSNRTSSGS
GGYIGIDCKTALACTATTEVVRKKRTGVITFRPSCRPACSPVSEISGSLMNRRKGTPHRAPL

H
>MtPLATZ14
MDTVLVPPWLEPLLKTPFFNICRIHADAARNECNMYCLDCNNGDGAFCFYCRSSRHKDH
QVIQIRRSSYHDVVRVSEIQKVLDISGVQTYVINSARVLFLNVRPQPKSGKGVAHICEICGR
SLLDSFRFCSLGCKLERIKKNGDASFALEGKNEGLTMEGVSSSRSEEELREGSTQDMYPLT
PPPPPLNARRRKGIPHRAPFGSSF
>MtPLATZ15
MAFENQDTVREIKPKNRRIMGAGGPDDEENRWPPWLKPLLKERFFVQCKLHADSHKSEC
NMYCLDCMNGALCSLCLNYHKDHRAIQIRRSSYHDVIRVNEIQKVLDITGVQTYIINSARV
VFLNERPQPRPGKGVTNTCEVCERSLLDSFRFCSLGCKIVGTSMNFQKKNKSARASMISDS
EDSHSSSCSHGRLKNIKVQSFTPSTPPPTVNYRSAKRRKGIPHRAPMGGLVLQY
>MtPLATZ16
MGCQKPSWLEALYTEKFFAGCSYHENAKKNEKNVCCLDCCTSICPHCFPSHRYHRLLQV
RRYVYHDVLRLEELEKLIDCTNVQAYTINSAKVVFIKKRPQNRQFKGSGNYCTSCDRILQE
PFIHCSLGCKVDFVLKHYKDLSPYLRICNTLQLSPDYLIPQEMGDELEMTRSTIVDCDDPM
SSYSVSSSGSENMSMPMPCTEIVRKRRSGWTSMCAKFMANSNSNKVSDEDMATSMISRR
KGVPHRSPLC

Supplementary Table S2 The *Ka/K*s ratios of the duplicated *MtPLATZ* gene pairs.

Gene 1	Gene 2	*K*a	*K*s	*K*a/*K*s
MtPLATZ1	*MtPLATZ9*	0.35	0.99	0.35
MtPLATZ2	*MtPLATZ14*	0.14	0.71	0.19
MtPLATZ3	*MtPLATZ7*	0.16	0.81	0.19
MtPLATZ6	*MtPLATZ10*	0.13	0.46	0.29
MtPLATZ11	*MtPLATZ15*	0.09	0.80	0.12

Supplementary Table S3 TMM values of *MtPLATZ* genes in different organs and developing seed tissues of *M. truncatula*

**Name**	**Root**	**Noudle**	**Shoot**	**Stem**	**Leaf**	**Flower**	**17 dap EM**	**26 dap EM**	**36 dap EM**	**44 dap EM**	**17 dap EN**	**26 dap EN**	**36 dap EN**	**44 dap EN**	**17 dap SC**	**26 dap SC**
MtPLATZ11	204	27.20	56.7	69.8	400	25.7	4.31	2.10	12.2	31.2	10.80	5.36	14	24.2	57	133
MtPLATZ15	539	405	38.7	502	877	11.6	1.03	0.871	5.23	80.4	10.30	2.43	15.8	85.5	37.7	113
MtPLATZ6	71.3	4.31	32.1	98.6	113	5.59	1.85	2.31	4.20	4.56	13.80	8.58	7.28	5.38	29.8	76.3
MtPLATZ10	13.5	0.20	0.92	7.28	3.23	0.26	0.10	0.05	0.40	0.20	4.41	1.46	0.28	0.36	0.49	0.18
MtPLATZ3	0.05	0.05	0.05	0.05	0.05	0.05	0.10	0.05	0.05	0.05	0.05	0.05	0.05	0.05	0.05	0.05
MtPLATZ4	0.05	0.10	0.05	0.05	0.05	0.05	0.05	0.05	0.05	0.05	0.05	0.05	0.05	0.05	0.05	0.05
MtPLATZ2	21.70	6.20	5.48	6.82	7.84	4.25	0.87	0.56	0.41	0.10	4.18	8.30	11.00	4.59	19.2	7.66
MtPLATZ14	17.4	2.67	9.40	4.10	1.69	7.48	1.79	1.38	3.79	1.54	9.15	9.35	14.6	14.6	1.49	0.72
MtPLATZ5	0.10	0.05	1.08	0.359	0.05	0.82	0.40	0.31	1.28	0.97	0.07	0.07	0.07	0.20	0.10	0.18
MtPLATZ7	0.10	0.05	0.20	0.05	0.05	0.05	0.05	0.05	0.05	0.05	0.05	0.05	0.05	0.05	0.05	0.07
MtPLATZ16	8.00	13.10	0.05	0.05	0.05	0.05	0.30	0.46	0.51	0.41	0.05	0.18	0.05	0.10	0.13	0.44
MtPLATZ13	0.05	0.05	0.20	0.05	0.05	0.20	0.05	0.05	0.05	0.05	0.05	0.08	0.05	0.05	0.05	0.05
MtPLATZ1	0.10	0.05	0.05	0.15	0.05	0.05	0.05	0.05	0.05	0.05	0.18	0.05	0.05	0.05	0.05	0.05
MtPLATZ9	13.22	2.92	3.72	6.61	11.2	5.13	7.43	2.82	2.36	1.13	15.20	11.30	5.56	2.31	11.6	8.25
MtPLATZ8	1.51	0.30	1.95	2.46	3.64	1.03	0.10	0.05	0.67	0.15	1.03	0.97	0.85	0.79	2.15	2.51
MtPLATZ12	1.74	1.85	0.95	1.49	1.23	1.18	0.10	0.10	0.36	0.31	0.56	0.26	0.23	0.38	1.69	1.49

Note: dap, days after pollination; EM, embryo; EN, endosperm; SC, seed coat.

Supplementary Table S4 The sequences of the primers used for the qRT-PCR analysis.

Gene name	Forward primer	Reverse primer
Mtactin	TGGATTCTGGTGACGGTGTG	CGGCTGAGGTGGTGAACAT
MtPLATZ2	ATGAGTGTCGGATTCACGCC	CAGCCACCCTCACTACATCA
MtPLATZ6	TGGGAGGTGGAGGAGGTAAC	AAACAGAACAGAGGGCTCCA
MtPLATZ9	CAAACCTCGGCCTCCAATCT	GGCTTTGAGGCTCCCCTATC
MtPLATZ10	TTCACCTACTGCTCCCTTGG	GCAATTTTGTAAGGTGGCGGT
MtPLATZ11	TTGGTTGCAAGATTGTGGGGA	TAACTGAAGTTGGGGGTGGT
MtPLATZ14	TGATGTGGTTCGTGTGTCAGA	TCCAACAAGCTCCTTCCACA
MtPLATZ15	ACTTGTGAAGTCTGCGAGCG	TGCTGCTGTGTGAATCCTCT
MtPLATZ16	CACCGCCTTCTTCAAGTTCG	TTGGCGGTTTTGAGGTCTCTT
